# Concurrent germline *BRCA1, BRCA2,* and *CHEK2* pathogenic variants in hereditary breast cancer: a case series

**DOI:** 10.1007/s10549-021-06095-w

**Published:** 2021-01-28

**Authors:** Jasmine Sukumar, Mahmoud Kassem, Doreen Agnese, Robert Pilarski, Bhuvaneswari Ramaswamy, Kevin Sweet, Sagar Sardesai

**Affiliations:** 1 Division of Medical Oncology, The Ohio State University Wexner Medical Center, 1204A Lincoln Tower, 1800 Cannon Dr., Columbus, OH 43210 USA; 2 Division of Human Genetics, The Ohio State University Wexner Medical Center, Columbus, OH USA; 3 Division of Surgical Oncology, The Ohio State University Wexner Medical Center, Columbus, OH USA

**Keywords:** BRCA1, BRCA2, CHEK2, DNAI1, Germline, Genetic, Variants, Breast cancer

## Abstract

**Background:**

Concurrent germline (g) pathogenic variants related to hereditary breast cancer represent a rare occurrence. While double heterozygosity in gBRCA1 and gBRCA2 has been reported in the past, herein we describe the first case of three known concurrent pathogenic variants identified in a family with a strong history of breast cancer.

Case presentation

The proband is a 55-year-old female diagnosed with synchronous bilateral breast cancers. She underwent a multi-gene panel testing indicating the presence of 3 concurrent heterozygous germline deleterious variants in *BRCA1* (*c.181T* > *G)*, BRCA2 (*c.4398_4402delACATT*), and CHEK2 (*1100delC*). The patient’s two daughters (34 and 29 years-old) were found to be transheterozygous for inherited pathogenic variants in BRCA1 *(c.181T* > *G)* and CHEK2 *(1100delC)* genes*.*

**Conclusion:**

The cancer risk and phenotypic manifestations associated with transheterozygous or multiple concurrent deleterious germline variants in hereditary breast cancer requires further investigation. A personalized approach to counseling, screening, and risk reduction should be undertaken for these individuals.

## Introduction

Germline (g) variants in breast cancer susceptibility genes are a significant risk factor for the development of breast cancer [1, 2] and as much as 10–15% of breast cancer cases are hereditary [3, 4]. This includes both rare, high penetrance variants (e.g., *BRCA*) and rare, moderate risk variants (e.g., *CHEK2*) [5, 6]. Clinical features associated with hereditary cancers such as early age of disease onset, family history of breast and ovarian cancer, bilateral breast cancer, male breast cancer, and Ashkenazi Jewish ancestry should prompt referral for genetic counseling [2, 7]. Understanding the precise risks and clinical implications associated with pathogenic variants is critical to establish best practices in screening and prevention and thereby improve outcomes for patients who are predisposed to hereditary breast cancer.

Of the known pathogenic variants related to hereditary breast cancer, those in the Breast Cancer Susceptibility Genes 1 and 2 (*BRCA1/2*) account for 50–60% of cases [1, 8]. Additionally, germline *BRCA1* pathogenic variants are more often associated with triple negative breast cancers with worse prognosis [9]. *BRCA1/2* pathogenic variants account for approximately 1–2% of all breast cancer cases and the prevalence of *BRCA1/2* pathogenic variants in the general population is about 1 in 400 individuals [10]. However, the prevalence in the Ashekanzi Jewish population is higher at about 1 in 40 individuals, or 2 to 2.5% [11, 12]. BRCA1/2 genes code for tumor suppressor proteins involved in DNA double strand break repair; these proteins play a critical role in response to maintenance of genetic integrity. Aberration of BRCA proteins involved in the DNA repair pathway leads to homologous recombination deficiency and diminished response to DNA damage, thereby contributing to carcinogenesis [13]. *BRCA1*/2 pathogenic variants demonstrate high penetrance with approximately 50–90% lifetime risk of breast cancer. In addition, *BRCA1/2* variants can increase the risk of second primary breast cancer [2, 14, 15] and other solid tumors such as high-grade serous ovarian cancers, pancreatic adenocarcinoma, and melanomas [14].

Hereditary breast cancer is also caused by pathogenic variants in other well-reported susceptibility genes such as *ATM, CDH1, CHEK2, PALB2, PTEN, TP53,* and *NF1. CHEK2* (cell cycle checkpoint kinase 2) is a moderately penetrant breast cancer susceptibility gene which can increase the breast cancer risk by two to threefold [16–18]. The prevalence of CHEK2 pathogenic variants in the general population is approximately 1% [5]. While deleterious CHEK2 mutations are reported at a higher frequency in individuals of Northern [19] and Eastern European descent [20], the exact prevalence in these populations compared with the general population is not clearly quantified. However, in one Polish study of 7494 breast cancer patients and 4346 controls, a CHEK2 pathogenic variant was detected in 3% of women with breast cancer and 0.8% of women in the control group [20]. The CHEK2 gene codes for a serine/threonine protein kinase called Chk2 kinase which activates multiple proteins critical in maintenance of the cell cycle and DNA repair. This includes the tumor suppressor protein p53 which has an integral role in cell cycle control and apoptosis. Additionally, Chk2 kinase activates the BRCA1 protein, and this interaction is important in the normal function of cellular DNA damage response [21, 22]. Therefore, CHEK2 and BRCA are closely interrelated in the DNA break repair pathway and work together to maintain genomic integrity. The most common variant in the CHEK2 gene is the frameshift mutation *1100delC* which codes for a truncated protein. This pathogenic variant is associated with a 28–37% lifetime risk of breast cancer, with the higher risk in those with strong family history [2, 20]. *CHEK2* pathogenic variants are also implicated in a 1.5–2 fold increase in colon cancer risk [23].

There is a paucity of data on the risk and clinical implications of an individual carrying multiple simultaneous pathogenic variants. The presence of co-existing *BRCA1/2* variants as well as concomitant *BRCA* and *CHEK2* variants in an individual is exceedingly infrequent but has been described in the literature [24–26]. However, to the best of our knowledge, there are no prior reports of three simultaneous pathogenic germline variants contributing to hereditary breast cancer and we describe the first reported case.

## Proband and family case presentation

The proband is a 55-year-old pre-menopausal Caucasian female with a past medical history of appropriately treated chronic hepatitis C infection, cirrhosis, coronary artery disease, and hyperthyroidism who was diagnosed with left breast cancer in August 2018. She underwent bilateral mastectomy and left axillary sentinel lymph node biopsy with bilateral reconstruction. Pathology revealed a 1.3 cm, grade 3 invasive ductal carcinoma which was estrogen receptor negative (0%), progesterone receptor weakly positive (10%), and Her-2/neu negative (IHC Score 0). She was pathologically staged as 1A (pT1cN0). An incidental right invasive ductal carcinoma was additionally found on review of the pathologic specimen, which was 0.3 cm, grade 1 and strongly hormone receptor positive (estrogen receptor 100%, progesterone receptor 100%) and Her-2/neu negative (IHC score 1+). Systemic adjuvant chemotherapy was recommended for the left breast cancer but the patient declined. She started adjuvant endocrine therapy with tamoxifen.

Notably, the proband endorsed a strong family history of breast cancer including a mother diagnosed at the age of 42 (bilateral, synchronous primaries), a sister at the age of 35, and a maternal grandmother at the age of 43. Family history was also significant for melanoma in her brother. There was no family history of ovarian cancer. To her knowledge, none of her relatives had previously completed genetic testing. The patient was referred to a licensed genetic counselor for cancer genetic risk assessment and pre-test genetic counseling. Blood-based multi-gene germline testing was performed using an 11-gene panel that included *ATM, BRCA1, BRCA2, CDH1, CHEK2, NBN, NF1, PALB2, PTEN, STK11,* and *TP53.* The results indicated presence of three concurrent heterozygous germline pathogenic variants in *BRCA1* (*c.181T* > *G)*, *BRCA2* (*c.4398_4402delACATT*), and *CHEK2* (*1100delC*). The proband received post-test genetic counseling and was advised on her cancer risk and risk reduction recommendations, as well as the potential inherited risk to her relatives. She elected to undergo laparoscopic bilateral salpingo-oophorectomy for ovarian cancer risk reduction, and pathology from this surgery was benign.

The patient’s 34-year-old daughter, a pre-menopausal female with a past medical history significant for bipolar disorder, was referred for genetic counseling for her 50% risk for each of the three separate pathogenic variants identified in her mother. There was no known family history of cancer in her father. She elected to pursue genetic testing for the 3 genes (*BRCA1, BRCA2, CHEK2*) which revealed a transheterozygous inheritance of pathogenic variants in the *BRCA1 (c.181T* > *G)* and *CHEK2 (1100delC)* genes*,* but did not report the *BRCA2* pathogenic variant identified in her mother*.* In October 2019, she had her first screening breast MRI and was found to have a suspicious mass in the right breast; subsequent biopsy revealed right invasive ductal carcinoma. She underwent a right radioactive seed-localized lumpectomy and sentinel lymph node biopsy in December 2019 demonstrating two foci of grade 3 invasive ductal carcinoma (0.7 cm and 0.25 cm) with negative margins and negative sentinel lymph nodes, estrogen receptor low-positive (5%), progesterone receptor negative (0%), and Her-2/neu negative (IHC score 1+). She subsequently received adjuvant chemotherapy. However, she declined adjuvant endocrine therapy due to concern for possible side effects. Almost a year from diagnosis, she underwent a risk-reducing bilateral skin sparing mastectomy followed by reconstruction.

**Fig. 1:**
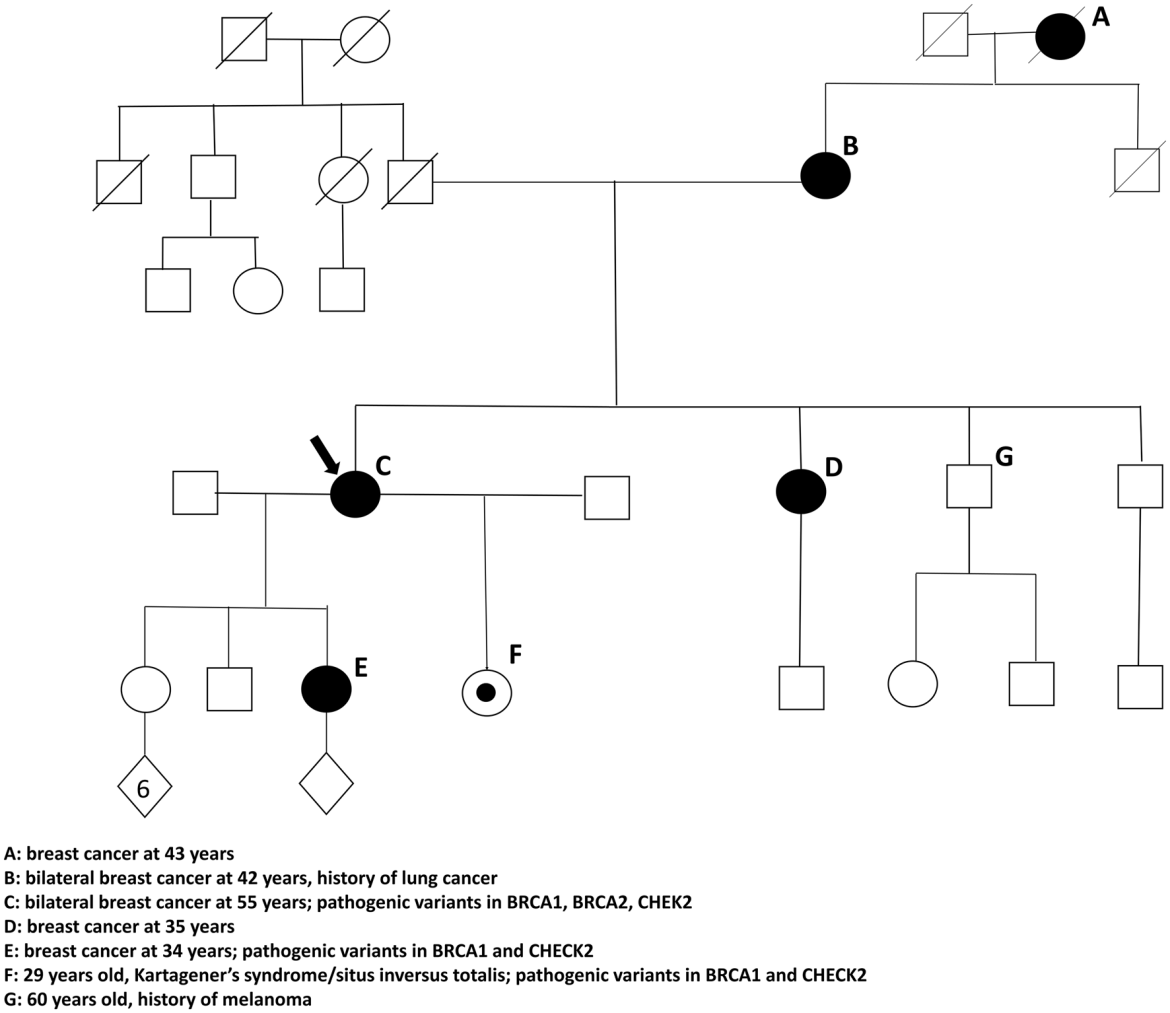
Family pedigree

Based upon the described family history and genetic results, the proband’s 29-year-old daughter underwent genetic testing. She had a different father than her 35-year-old half-sister, and to the proband’s knowledge, there was no paternal family history of cancer. The results of genetic testing demonstrated the inheritance of pathogenic variants in BRCA1 *(c.181T* > *G)* and CHEK2 *(1100delC)* genes. She has since established care in our institutional high-risk breast cancer program to undergo annual bilateral screening mammograms and bilateral breast MRIs. She declined chemoprophylaxis with endocrine therapy. A family pedigree is provided in Fig. 1.

## Discussion

We describe the first reported case of three concurrent pathogenic germline variants in *BRCA1*, *BRCA2*, and *CHEK2* associated with inherited breast cancer in an individual. Given the presence of 3 co-existing pathogenic variants in the proband, her family members were at a very high risk of carrying at least one moderate or high penetrance variant. Upon further genetic testing, we identified co-existing germline *CHEK2* and *BRCA* pathogenic variants in this family cluster with breast cancer; this represents a rare occurrence with only few reported cases.

In review of the literature, including case reports [25–27] as well as public registries of human genetic variants [24, 28], simultaneous pathogenic variants in two different genes (referred to as double heterozygosity or transheterozygosity) related to hereditary breast cancer is an extremely uncommon occurrence. For example, there have been less than 100 reported cases of co-existing *BRCA1/2* variants; this is primarily in the Ashkenazi Jewish population, among whom *BRCA* pathogenic variants are ten times more common than in people not of Jewish descent [24, 27–34]. A retrospective study by Rebbeck et al. reported *BRCA1/2* double heterozygosity in 93 (0.3%) of 32,295 *BRCA* 1/2 germline pathogenic variant carriers. Of these, 64 patients were diagnosed with breast cancer and 17 patients with epithelial ovarian cancer. Double heterozygotes were more likely to be diagnosed with breast cancer compared to women with only one deleterious *BRCA* pathogenic variant (68.1% vs. 52.0% for BRCA1, *p* = 0.002; 67.4% vs. 50.4% for BRCA2, *p* = 0.002). Furthermore, this study demonstrated that patients with two inherited pathogenic variants were more likely to develop ovarian cancer compared with those with a *BRCA2* pathogenic variant alone (16.9 vs. 9.3%, *p* = 0.0017) [25].

In another retrospective study of a Jewish population of 1191 carriers of *BRCA* 1 and 2 variants, 22 patients (1.8%) were double heterozygotes. This investigation suggested a slightly younger age of cancer diagnosis but a similar probability of breast and ovarian cancer development in double heterozygotes compared with single variant carriers [34]. In a study by Leegte et. al, the rate of *BRCA1/2* double heterozygosity was 0.36% in *BRCA* variant carriers; this rate increased to 1.8% in the sub-population of Askhenazi Jewish patients. In the 34 women with double heterozygosity, there was no significant phenotype differences compared with single variant carriers [27]. Simultaneous *BRCA1/2* pathogenic variants were also identified in a systematic review of women with epithelial ovarian cancer. The clinical and prognostic features of individuals with double heterozygosity closely resembled *BRCA1* variant carriers [29]. In summary, due to the infrequent number of reports, there is wide variability and limited clarity of the clinical and prognostic features associated with having two deleterious germline variants in *BRCA*.

Double heterozygosity in other breast cancer susceptibility genes has also been reported but represents an even rarer occurrence. Relevant to this case study, there have been only a handful of reports of co-existing *CHEK2* and *BRCA* variants. For example, an investigation of germline variants in 5,391 Slavic patients with breast cancer found 17 breast cancer patients in which two pathogenic variants were detected; 4 (1.4%) of these women were found to be *CHEK2* and *BRCA1* double heterozygotes. The *CHEK2* variants included 2 patients with *CHEK2 del5395,* 1 patient with *CHEK2 1100delc* and 1 patient with *CHEK 2 IVS2* + *1G* > *A.* Additionally, one patient in the matched group of 3,884 healthy controls without breast cancer was found to harbor a variant in both *CHEK2* and *BRCA1* [35]. In another study by Turnbull et al., *CHEK2 1100delC* was found in 1/358 (0.28%) of those with *BRCA1* pathogenic variants and 1/357 (0.28%) *BRCA2* pathogenic variant cases [26]. Additionally, co-existing *BRCA1* with *CHEK2* cases was reported in analysis of a large Polish cohort (7,782 breast cancer and 6,233 control cases), in which patients were assessed for seven founder pathogenic variants in *BRCA2* and *CHEK2*. Double heterozygosity was detected in 15 patients in the breast cancer group and 2 patients in the control group [36]. Conclusions from each of the described case studies of co-existing *CHEK2* and *BRCA* variants suggest no significant increased cancer risk or difference in phenotype beyond what is expected with a single *BRCA* variant [26, 35, 36]. However, this requires further verification given the limited number of such cases.

Evidenced-based management of individuals with confirmed high penetrance pathogenic variants such as BRCA1 and BRCA2 can result in earlier detection of breast cancer when early and timely screening using optimal detection methods is applied. Additionally, chemoprophylaxis with endocrine therapy and surgery could result in risk reduction when optimally applied [37–40]. Specifically, risk-reducing bilateral mastectomy has been shown to reduce breast cancer development up to 90% [39]. Additionally, bilateral salpingo-oophorectomy, typically after completion of child-bearing and between the age of 35–40 with *BRCA1* variants and 40–45 with *BRCA2* variants, reduces the risk of ovarian cancer by as much as 80% and has a mortality benefit [41]; it may also reduce the risk of breast cancer, although these data are inconsistent [41, 42]. For *CHEK2*, current guidelines recommend screening with annual mammography with consideration of annual breast MRI starting at the age of 40. Additionally, more intensive colon cancer screening is indicated with specific recommendations based on family history of colon cancer [40]. However, there is insufficient evidence to recommend other risk reduction strategies such as bilateral mastectomy, and decision-making should be based on individualized patient and family risk [40].

Decades of research regarding the risk and phenotypic manifestations associated with individual germline variants in hereditary breast cancer has led to the development of informed clinical practice guidelines [2, 40]. However, a challenging aspect of personalized counseling for *BRCA1/2* as well as *CHEK2* pathogenic variants is the relatively large range of cancer risk [14, 36]. This represents an even greater challenge in those with multiple pathogenic variants. In these individuals, the degree of risk is unclear based on the limited reported cases and there are challenges in interpreting management recommendations which apply to single variants. Greater data are needed to understand the clinical implications of an individual harboring multiple variants, such as age of cancer onset, primary tumor characteristics and possibility of multiple primary tumors. A better understanding of the cumulative lifetime cancer risk and whether there is a potential synergistic effect of multiple pathogenic variants on carcinogenesis is additionally necessary. Furthermore, it is unclear how other genetic factors such as polygenic risk scores could further modify risk in individuals with co-existing variants, and whether there is resemblance to which these factors impact those with single pathogenic variants [43]. A better understanding of this risk is critical to establish informed clinical decisions and best practices in counseling, screening, risk reduction, and treatment of those with co-existing variants. For patients with double or multiple heterozygosity in known cancer-predisposing genes, our institutional practices consider the highest risk gene variant to guide relevant screening or prevention strategies. For instance, the proband in this case was counseled on breast and ovarian cancer risk based on the presence of a known *BRCA1* variant. However, she also received counseling for management of risk for other malignancies including colon cancer, pancreatic cancer, and melanoma based on the presence of concurrent pathogenic mutations in *BRCA2* and *CHEK2*.

Based on individualized care and shared decision-making, certain patients with co-existing variants may benefit from more intensive surveillance/screening programs or risk reduction strategies beyond the standard recommendation for single pathogenic variants. It will also be critical to understand whether such interventions may affect survival in this population. Additionally, biologic data on the risks associated with multiple germline variants may influence decisions related to therapeutics, such as use of platinum-based chemotherapy or PARP inhibitors, in those diagnosed with breast and ovarian cancer. Namely, it is unclear whether the efficacy of these DNA damaging agents will be impacted by multiple variants in comparison to those with a single deleterious variant.

This family case report also emphasizes the importance of multi-gene panel testing as the preferred method of genetic analysis in patients at risk for hereditary breast cancer, which is the current standard of care for genetic assessment in the proband. This strategy not only improves efficiency and cost-effectiveness, but may also detect multiple susceptibility genes in an affected individual, as was seen in our case report. Moreover, in individuals known to carry a single pathogenic variant based on phenotype-directed testing, a detailed family and cancer history should be obtained. In these situations, variant analysis should be extended on a case-by-case basis in those with a strong clinical suspicion of manifesting multiple pathogenic variants, such as a strong family history of cancer on both sides. In this family case series, single-site genetic testing for the pathogenic variants found in the proband was pursued in the offspring as there was no known paternal family history of cancer in the offspring. These recommendations are in concordance with the NCCN guidelines [40]. This may impact clinical management as knowledge of multiple susceptibility gene variants in an individual may guide tailored recommendations and modifications in cancer screening and risk reduction approaches.

## Conclusion

Identification of more individuals with multiple co-existing germline variants related to familial breast cancer will facilitate an improved understanding of the phenotypic manifestations of these unique genotypes in family clusters and provide better insight into optimal clinical management practices. There is currently no standard of care for management in patients with co-existing pathogenic variants, and a personalized and tailored approach in genetic counseling, early detection, and risk reduction strategies should be undertaken with the goal to improve cancer and health-related outcomes.

## Data Availability

All data are included in the manuscript.
